# Models of pancreatic ductal adenocarcinoma

**DOI:** 10.1007/s10555-021-09989-9

**Published:** 2021-09-07

**Authors:** Rayane Dennaoui, Hridaya Shrestha, Kay-Uwe Wagner

**Affiliations:** Department of Oncology, Wayne State University School of Medicine and Tumor Biology Program, Barbara Ann Karmanos Cancer Institute, 4100 John R, EL01TM, Detroit, MI 48201 USA

**Keywords:** Pancreatic cancer, Adenocarcinoma, Metastasis, KRAS, MYC, Dormancy

## Abstract

Although pancreatic cancer remains to be a leading cause of cancer-related deaths in many industrialized countries, there have been major advances in research over the past two decades that provided a detailed insight into the molecular and developmental processes that govern the genesis of this highly malignant tumor type. There is a continuous need for the development and analysis of preclinical and genetically engineered pancreatic cancer models to study the biological significance of new molecular targets that are identified using various genome-wide approaches and to better understand the mechanisms by which they contribute to pancreatic cancer onset and progression. Following an introduction into the etiology of pancreatic cancer, the molecular subtypes, and key signaling pathways, this review provides an overview of the broad spectrum of models for pancreatic cancer research. In addition to conventional and patient-derived xenografting, this review highlights major milestones in the development of chemical carcinogen-induced and genetically engineered animal models to study pancreatic cancer. Particular emphasis was placed on selected research findings of ligand-controlled tumor models and current efforts to develop genetically engineered strains to gain insight into the biological functions of genes at defined developmental stages during cancer initiation and metastatic progression.

## Introduction

Pancreatic cancer is currently the third-leading cause of cancer-related deaths in the USA, and without any considerable breakthroughs in early detection and treatment, this malignancy might become the second most lethal cancer type by the end of this decade [1]. Owing to this unfortunate trajectory, pancreatic cancer will undoubtedly be a major health issue in the USA and many other industrialized nations. More than 95% of pancreatic cancers arise in the exocrine compartment of this glandular organ, and pancreatic ductal adenocarcinoma (PDAC) is the most common type among pancreatic neoplasms. Despite an incremental increase in the 5-year survival rate from 6 to approximately 10% over recent years, PDAC remains to be a cancer type with a very dismal prognosis (NCI, SEER, Cancer Stat Facts: Pancreatic Cancer). The absence of defined symptoms and the lack of clinically validated biomarkers and practical imaging modalities suitable for early detection in the general population are all contributing factors that around 50–60% of pancreatic patients present with metastatic disease at the time of diagnosis. The surgical resection of a primary tumor remains to be the only curative therapy for PDAC, but only about 10–20% of patients are amenable to this procedure. The survival rate of patients with resectable early-stage tumors is about 39% but drops sharply if the cancers are locally advanced (American Cancer Society’s Cancer Facts & Figures, 2021). Systemic treatment with FOLFIRINOX and nab-paclitaxel/gemcitabine has been adopted as first-line therapy regimens to treat metastatic disease, but these drugs are very toxic and may not be tolerated in a subset of pancreatic cancer patients with cachexia. The median overall survival of patients receiving these drugs does not exceed 12 months [2–4].

The onset and progression of pancreatic ductal adenocarcinoma occur as a consequence of inherited and somatically acquired mutations, the epigenetic deregulation of genes, and changes in the post-translational modification of proteins that play key roles in neoplastic transformation, local invasion, and metastasis. A multitude of new methodologies made it possible to delineate the genome, transcriptome, and proteome of pancreatic cancers over the past decade and provided a detailed insight into the molecular and developmental processes that govern the genesis of pancreatic cancer. These approaches, along with the latest technological advances in the large-scale analyses of single cells within tumors, illuminated the intertumoral and intratumoral heterogeneity of PDAC. Compared to other tumor types such as breast cancer, the classification of PDACs into molecular subtypes is at an early stage and lacks clinical applicability. Nonetheless, these efforts have led to the identification of crucial drivers and potentially actionable targets in certain subsets of PDAC. To validate the biological significance of putative new targets that are identified with various omics approaches and to understand the molecular mechanisms by which they contribute to the evolutionary processes of PDAC, there is a continuous need for the development and analysis of preclinical and genetically engineered pancreatic cancer models. Following an introduction into the genetic events that drive PDAC, this review will highlight selected recent advances in genetic modeling to study the etiology of this malignancy.

## Molecular subtypes of PDAC

Among premalignant lesions with distinct histopathological features, pancreatic intraepithelial neoplasia (PanINs) are the most common precursor lesions that are associated with the development of invasive PDAC. The resulting malignant tumors may divert into morphological variants such as adenosquamous, colloid, medullary, hepatoid, micropapillary, or undifferentiated carcinoma that exhibit differences in the biological behavior and prognosis [5]. Over the past decade, there has been significant progress to stratify PDACs into molecular subtypes that may be of clinical relevance. Compared to other human malignancies such as breast cancer, the subclassification of PDAC is less defined. The designation of PDAC subtypes is based on findings using different approaches as well as the inclusion of molecular features of the tumor-associated stroma. The microarray-based analysis of the transcriptomes of untreated PDAC specimens as well as established cancer cell lines conducted by Collisson et al. led to a stratification of PDAC into three molecular subtypes based on a 62-gene signature: classical, quasi-mesenchymal (QM), and exocrine-like subtypes [6]. Compared to the classical subtype, patients with tumors that showed quasi-mesenchymal characteristics had a poorer prognosis, and cancer cells with these molecular features exhibited a variable response to therapeutic drugs. Specifically, the QM subtype seems to be more sensitive to gemcitabine, whereas the classical subtype showed a better response to erlotinib. Interestingly, PDACs of the classical subtype displayed a higher expression of GATA6, which is an important transcription factor required for normal pancreatic organogenesis [7, 8]. Using virtual microdissection of transcriptomic analyses of primary and distant metastatic tumors, Moffitt and coworkers characterized two main tumor-specific subtypes of PDAC: classical and basal-like [9]. Additionally, they discriminated cases based on the presence of a “normal” or an “activated” tumor-associated stroma. The “classical” subtypes in both transcriptomic analyses show similarities in the expression of GATA6 and extracellular mucins. In contrast, basal-like PDACs predominantly express laminins and keratins that are found in the basal subtypes of bladder and breast cancers [10, 11]. Moreover, patients with the basal-like subtype had a significantly worse overall median survival of 11 months when compared to 19 months in patients with the classical subtype. An important biological insight from the study by Moffitt et al. is that metastatic lesions often retain tumor-specific signatures of the primary pancreatic tumor indicative of a low within-patient heterogeneity [9].

Using an array-based mRNA expression profiling approach, Bailey et al. [12] stratified pancreatic cancers into four subtypes based on the differential expression of ten transcription factor networks: squamous, pancreatic progenitor, immunogenic, and aberrantly differentiated endocrine exocrine (ADEX). These four subtypes showed some correlation to tumors with specific histopathological features. Specifically, the squamous subtype, which served as an independent factor for poor prognosis, exhibited a characteristic expression of genes within molecular networks that are involved in inflammation, TGF-β signaling, as well as c-MYC and TP63 and their transcriptional targets. Using the respective clustering algorithms from the previous publications [6, 12], mRNA expression profiling conducted under the auspices of the Cancer Genome Atlas Research Network [13] was able to confirm the aforementioned 4-group, 3-group, and 2-group classifications. Then again, the findings of this study suggested that the molecular subtyping might be profoundly affected by the purity of tumor samples and transcripts from non-neoplastic cells. The collective results of the study showed that tumor specimens with a high purity fall into two distinctly different molecular subtypes: a basal-like/squamous type and a classical/progenitor type. This 2-subgroup molecular classification of pancreatic cancer is currently receiving broad recognition [14].

Similar to the transcriptome analyses, the whole-exome sequencing study conducted by Witkiewicz et al. demonstrated that selected mutations in PDAC patients were associated with clinical prognosis and histopathological subtypes [15]. While loss-of-function mutations in the chromatin remodeling gene *ARID1A* conferred poor outcome, mutations in *RBM10*, a regulator of alternative splicing, were present in specimens of patients with extended survival. When the team applied bioinformatics approaches to interrogate regions in the genome that are significantly amplified or deleted across PDAC cases, they found that amplifications within chromosome 8q24 containing the *c-MYC* oncogene were highly associated with poor outcomes. Moreover, the amplification of *c-MYC* was significantly overrepresented in the adenosquamous pathological subtype. The higher incidence in *c-MYC* amplifications in adenosquamous carcinoma was validated in a recent study by Lenkiewicz et al. [16]. Moreover, the biological contribution of c-MYC to this aggressive form of pancreatic cancer was confirmed by the analysis of adenosquamous characteristics of pancreatic tumors that arose in a c-MYC-induced genetically engineered mouse model of PDAC that our team generated [17]. In addition to the similarities in histopathological features, these mouse tumors expressed TP63 which is a known marker of squamous differentiation in human PDAC [15].

In contrast to human breast cancers where the molecular profiles of the majority of tumors align with the expression of therapeutically relevant steroid hormone and growth factor receptors (i.e., ER, PR, and ERBB2), the two PDAC molecular subtypes can coexist in the same primary tumor and might be a phenotypic consequence related to mutational and epigenetic changes that promote the plasticity of cancer cells [18–20]. To elevate the clinical relevance of the proposed molecular subtypes, there is a need to better integrate the gene expression data and specific mutations with histopathological characteristics. Recent studies provide supporting evidence that there is a prognostic relationship between molecular and morphological parameters [21]. The molecular subtypes may also be increasingly important to better characterize available models to study pancreatic cancer such as cell lines, patient-derived organoids, xenografts (PDX), and genetically engineered animal models. This appears to be particularly critical when the gene expression profiles of the tumor-associated stroma are being used to stratify the molecular types of PDX models to assess their growth rates in mice in association with patient biology as done in the study by Moffitt et al. [9]. The authors reported that the basal-like PDACs show better engraftment and faster growth rates compared to the classical subtype, and the majority of PDX had an activated stromal signature coming from the mouse regardless of the human cancer cell-intrinsic subtype. We will summarize in a later section of this review the intrinsic deficiencies of xenograft models such as the incompatibility of growth factors and their receptors between humans and mice that can profoundly affect the success of the engraftment of a primary tumor and promote the selection of cancer cells with particular molecular signatures.

## Key signaling pathways in pancreatic cancer

The limited number of validated molecular subtypes and the coexistence of tumor cells with both molecular signatures in the same patients are likely a consequence that most PDACs are driven by few somatic mutations that occur at high frequency (i.e., *KRAS*, *CDKN2A*, *TP53*, and *SMAD4*). A recent review by Hayashi et al. [14] provides a comprehensive overview of genetic abnormalities in pancreatic cancer and the contribution of high-frequency mutations as well as less common genetic alterations within core signaling pathways that drive the onset and progression of PDAC. Activating mutations in *KRAS* are present in more than 92% of PDAC cases [22, 23]. As single-nucleotide substitutions in codon 12 and, to a lesser extent, in codons 13 and 61 are present in low-grade PanIN lesions, it is evident that gain-of-function mutations in this small GTPase are common initiating events in pancreatic tumorigenesis [24, 25]. The important role of the MAP kinase pathway in neoplastic transformation is also evident in the roughly 10% of pancreatic cancers that lack oncogenic *KRAS* but carry mutations in upstream receptor tyrosine kinases (*FGFR1*, *ERBB2*), downstream effectors of RAS (*BRAF*), or loss of a negative regulator of active RAS (*NF1*) [13, 15]. The biological significance of mutant KRAS and sustained signaling of the MAP kinase pathway is not restricted to the initiation phase. An allelic imbalance of *KRAS* and additional mutations in regulators and effectors of the MAP kinase pathway increase the signal strength that is associated with cancer progression [26, 27]. This may explain why several of the human pancreatic cancer cell lines that are commonly used in research carry two mutant *KRAS* alleles (e.g., AsPC-1, MIA PaCa-2, Capan-1, KP-3; source NCI RAS Initiative). The allelic imbalance is not unique to pancreatic cancer and was also observed in a triple-negative breast cancer (TNBC) model that develops claudin-low (i.e., mesenchymal-like) mammary tumors in response to a mammary epithelial-specific activation of KRAS^G12D^ under the control of the endogenous KRAS locus [28]. Notably, the most commonly used human TNBC cell line to study metastatic breast cancer, MBA-MDA-231, carries mutations in *KRAS*, *BRAF*, and *NF1*, underscoring the universality of an increase in RAS signaling strength during the progression of diverse cancer types.

PanIN precursor lesions with a mutation in KRAS will not develop into a frank malignancy unless they acquire additional genetic abnormalities. Inactivation of the *CDKN2A* tumor suppressor locus, which encodes two proteins that control the cell cycle, INK4A and ARF, is closely linked to KRAS-associated neoplastic transformation [29, 30]. Ninety percent of all PDAC cases possess genetic (deletion, mutation) or epigenetic alterations (hypermethylation) in *CDKN2A* that mostly affect the expression of the INK4A protein, which is a critical regulator of the CDK4/6-Cyclin D-RB pathway. Since 80% of PDACs also acquire mutations in *TP53*, there seems not to be a stringent requirement for a loss of the *CDKN2A*-encoded ARF protein [30, 31]. Deficiency in TP53 has a pleiotropic effect on cellular processes other than cell cycle control that accelerate tumor progression, including genome instability, suppression of apoptosis, and altered metabolism [32]. Approximately half of all PDACs also acquire inactivating mutations in *SMAD4*, a crucial downstream effector of TGFβ signaling. Mutations in this pathway are suggested to play critical roles in invasion and metastasis [33].

Although it is known that c-MYC is a crucial downstream effector of oncogenic KRAS in other tumor types [34, 35], the biological importance of this transcription factor in PDAC had not been acknowledged until recently. It is particularly gratifying to us to see that c-MYC is now being included in the list of the most commonly altered genes in the genetic progression model of pancreatic cancer [14]. In a paper that was first submitted for publication in December of 2011, we reported that nuclear c-MYC is present in high-grade PanIN lesions and a significant subset of PDAC cases in both humans and mice [17]. Levels of c-MYC were also significantly higher in all human PDAC cell lines irrespective of the KRAS mutation status. Interestingly, we observed the highest expression of the c-MYC protein in BXPC-3 cells that carry wild-type *KRAS* alleles. Although we were able to demonstrate in that study that overexpression of c-MYC in transgenic mice was sufficient to initiate the stepwise developmental process of PanIN lesions and invasive PDAC with a high propensity to metastasize, our publication was met with harsh criticism stating that c-MYC had not been shown to play any role in PDAC and a model without mutant KRAS does not mimic the human disease. In contrast to that critique, it was subsequently shown that the transgenic c-MYC overexpression model appropriately recapitulated the squamous differentiation process that was observed in more aggressive human PDAC cases with amplifications in c-MYC [15]. The applicability of c-MYC as a marker for a dismal prognosis of pancreatic cancer has also been validated and can be attributed, in part, to elevated expression of this transcription factor in the basal subtype of PDAC that lacks GATA6 [36–39]. Several recent publications have provided mechanistic insight into the significance of c-MYC as an essential downstream node of RAS signaling in pancreatic cancer and other malignancies [40–42]. The widespread transcriptional changes that are controlled by c-MYC are central for the pleiotropic effects of mutant KRAS and other oncogenic pathways on protein synthesis, tumor cell growth, differentiation, metabolism, angiogenesis, and the suppression of the host immune response (for references, please refer to a review by Hessmann et al. [43]).

Besides the key signaling pathways that play pivotal roles in the initiation and progression of PDAC, there are a number of somatic alterations that occur at a lower frequency. These mutations may mainly contribute to the developmental trajectory of the histopathological and molecular subtypes of PDAC. Specifically, mutations in *GNAS* and *RNF43* that cause cystic premalignant lesions are present in about 10% of PDAC cases [29, 44, 45]. This suggests that a subset of PDAC cases may arise from preneoplasms other than PanINs. Unlike the well-defined roles of the major four recurrent mutations that cause sporadic PDAC, the list of infrequent germline mutations that are associated with hereditary forms of pancreatic cancer is continuously expanding [46]. Most germline mutations in patients with a familial history of PDAC were identified in tumor susceptibility genes that cause genome instability and impaired DNA repair mechanisms such as *PALB2*, *BRCA1/2*, *ATM*, and *MLH1/2/6*, as well as in *CDKN2A* and *TP53* [47–52]. For a disease like PDAC that currently lacks targeted therapies, some of these mutations provide new opportunities for treatment with platinum compounds, mitomycin C, and PARP inhibitors [53]. A detailed analysis of gene variants of unknown significance in ethnically diverse patient cohorts with familial cancer syndromes, including pancreatic cancer, might reveal additional germline mutations that contribute to an increased risk of developing PDAC.

## Inflammation and pancreatic cancer

An introduction into the etiology of pancreatic cancer would not be complete without highlighting the importance of inflammatory signals for the initiation and progression of PDAC. The discovery of an association between inflammation and cancer dates back to the seminal contributions of Rudolf Virchow more than 150 years ago and is nearly as old as the paradigm that cancers originate from normal cells [54, 55]. More recent epidemiological data and experimental findings provide clear evidence that inflammation is also an integral part of neoplastic progression in PDAC. Chronic pancreatitis increases the risk of developing pancreatic cancer [56], and the significance of environmental factors that cause chronic inflammation (e.g., smoking, heavy alcohol consumption, diet, and obesity) in pancreatic cancer are well documented [57–59]. It has also been reported that mutations in *PRSS1*, *SPINK1*, and *CFTR* that are linked to hereditary forms of pancreatitis increase the likelihood of developing PDAC [60–65]. In a mouse model for pancreatic cancer, Guerra et al. [66] have reported that the induction of chronic pancreatitis in adult animals with the cholecystokinin analog caerulein promotes the development of PanINs and ductal adenocarcinoma. The neoplastic process was accompanied by intralobular and interlobular-mixed inflammatory infiltrates (i.e., immune cells) and the formation of the desmoplastic stroma. On a mechanistic level, the study by Guerra et al. did not specifically implicate NF-κB signaling, a pivotal mediator of inflammatory responses, in mutant KRAS-induced PDAC progression, but Ling and coworkers [67] later demonstrated in a genetically engineered mouse model that oncogenic KRAS leads to a constitutive activation of NF-κB through IL-1α and p62. Remarkably, the deletion of IKK2/β in the pancreata of mice expressing mutant KRAS was sufficient to block the development of PanIN lesions and pancreatic tumors. The collective results led the authors to propose that cancer cell-intrinsic inflammatory signaling networks create a protumorigenic microenvironment through the expression of cytokines that facilitate angiogenesis and the recruitment of immune and stromal cells.

Among the cytokines that were deregulated by the ablation of NF-κB signaling in the study by Guerra et al. were several interleukins, including IL-6. Interleukin-6-class cytokines (e.g., IL-6, LIF, OSM, IL-11) are among few growth factors that are considered master regulators of cancer-associated inflammation [68]. All IL-6-class cytokines signal through specific ligand-receptor complexes that share the glycoprotein 130 (gp130) signal-transducing subunit, which activates Janus tyrosine kinases (JAKs) and downstream signal transducers and activators of transcription (STATs). A common characteristic of many cancers, including PDAC, is the persistent phosphorylation of STAT3 on Tyr705, which mediates the nuclear translocation and functionality of STAT3 as a transcription factor. It has been previously reported that STAT3 phosphorylation is a critical early event in the formation of precursor lesions for pancreatic cancer [69–71], and the conditional deletion of the *Stat3* gene in mice expressing mutant KRAS in the pancreas blocked the development of PanINs. More importantly, targeting STAT3 with shRNA constructs in ex vivo transformed cells that are deficient in *Trp53* reduced the formation of secondary tumors upon re-transplantation into recipient mice [70]. These collective findings may suggest that targeting the activation of STAT3 is a suitable strategy to prevent the initiation and progression of pancreatic cancer. This notion is supported by recent work from Shi et al. who demonstrated that the pharmacologic inhibition of LIF or the deletion of its receptor significantly slowed the progression of mutant KRAS-associated PDAC [72]. While this work highlighted a paracrine role of LIF produced by stellate cells on cancer cell differentiation and epithelial-mesenchymal transition, it should be noted that pancreatic cancer cell lines express active STAT3 [70]. Therefore, the persistent activation of STAT3 in cancer cells in vivo is likely a result of the combined action of IL-6 class cytokines that function in a paracrine and in an autocrine manner as shown by Fukuda et al. [71]. In sharp contrast to the more widely accepted view that STAT3 promotes tumor progression, there are also reports that this transcription factor may serve as a tumor suppressor that facilitates differentiation and an epithelial identity of cancer cells [73, 74]. It is currently unknown whether JAKs and STATs can have opposite biological roles in normal and neoplastic cells in the pancreas, which is a phenomenon that has been studied in other secretory tissues such as the mammary gland. There, active JAK1 and STAT3 accelerate the removal of secretory epithelial cells in response to locally produced inflammatory cytokines during post-lactational tissue remodeling [75–77]. Despite the known significance of active STAT3 in PDAC progression, limited progress has been made in the development of STAT3-specific inhibitors. To apply pharmacological agents that target STATs and their upstream JAKs for the prevention and treatment of pancreatic cancer, the biological significance of these transducers of inflammatory cytokines in normal pancreas development and PDAC progression still needs to be investigated in more detail.

## Xenograft modeling

**Fig. 1:**
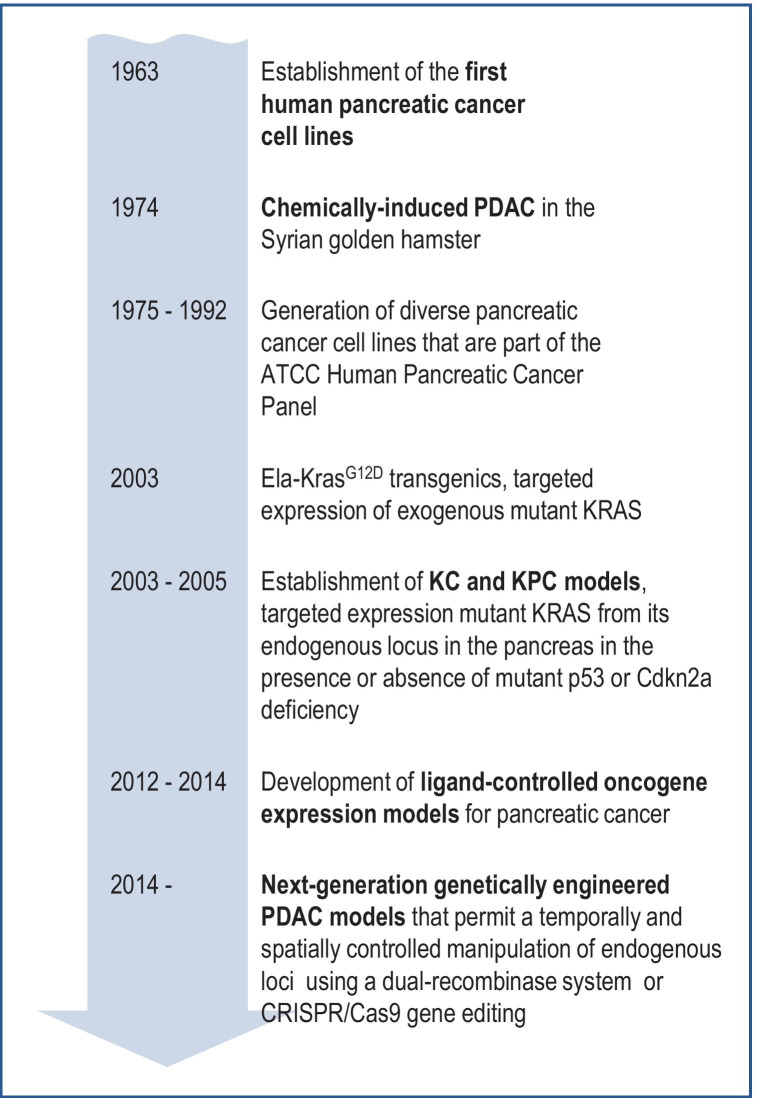
Milestones in the development of experimental models for pancreatic cancer

The establishment of the first pancreatic cancer cell lines dates back to 1963 [78] (Fig. 1). Over the past 60 years, many laboratories built an extensive arsenal of lines, but only a limited number are being routinely applied in biomedical research [79]. Interestingly, most of these commonly used pancreatic cancer cell lines, which have been in service for nearly four decades, were derived from metastatic sites and fewer came from primary pancreatic tumors (e.g., AsPC-1, Capan-2, BxPC-3). Authenticated reagents are available from the American Type Culture Collection (ATCC), and these cell lines have been invaluable tools for molecular and cellular experiments and drug development. A discourse on the applicability of human pancreatic cancer cell lines as preclinical models in cancer research as well as their shortcomings for clinical translation can be found in a review by Hwang et al. [80]. Despite the widespread use of well-characterized cancer cell lines, it should be noted that there is still a limited availability and insufficient applicability of genetically diverse, untransformed pancreatic cell culture models such as the hTERT-HPNE line generated by Lee et al. [81] in experimental research as well as the screening of chemical compounds.

The commonly used pancreatic cancer cell lines have been demonstrated to form tumors when transplanted into immunocompromised mice. The take rates, the speed of tumor formation, and the propensity to metastasize may vary considerably among the lines and are dependent on the type of transplant model (orthotopically or ectopically), the route of cell delivery (e.g., in situ implantation, intra-abdominal, subcutaneous, or intravenous injection), and, to some degree, the genetic background of the immunodeficient host (Athymic Nude, SCID) [82–84]. A table summarizing the growth properties of human PDAC cell lines in vivo is provided in a review by Kong et al. [85]. For many years, cell line-based xenograft models have been essential tools for preclinical research and to study complex biological processes such as metastasis in vivo. Notable improvements in xenograft modeling have been achieved by establishing patient-derived cancer transplant models (PDX), thereby, bypassing the need for cancer cells to adapt to cell culture conditions [82, 86]. PDX models may, at least initially, retain the original cellular heterogeneity and clonal diversity. They hold the promise to better resemble the properties of the primary tumors from which they were derived and, consequently, they are suggested to more accurately mirror therapeutic responses [87–89]. PDX models are applicable in research projects related to molecular therapeutics and for proof-of-principle experiments in “chemical biology” where pharmacological agents are being applied to examine their effects on cellular processes.

PDX models are less suitable as research tools for basic science and mechanism-oriented research studies that apply methods to manipulate genes in specific cell types. Even the use of the latest technologies in gene editing would require the dissociation of patient-derived tumors into their cellular components to validate the correct targeting events. In a commentary published in 2004, we listed five major shortcomings of PDX models that are similar to cell line-based conventional xenografts [90]. Some of these limitations also apply to patient-derived tumor organoid models. First and foremost, PDX models are derivatives of an established malignancy, and therefore, they are unsuitable to study the roles of genes and molecular processes in normal tissue homeostasis and disease initiation. Second, all xenograft models are conducted in immunocompromised hosts that lack a normal immune response against tumor cells. Third, the human stromal cells within an engrafted tumor fragment are swiftly replaced by cells from the host, and cancer cells engage exclusively with murine cell types at metastatic sites. Fourth, all heterogeneous cancer cell populations within a tumor, regardless of whether they are propagated in vivo or in culture, will be subject to genetic drift. In support of this premise, Ben-David et al. recently provided experimental evidence that, based on the dynamics of copy number alterations (CNAs), PDX models undergo a mouse-specific tumor evolution [91]. CNAs may not serve as the only indicator for genetic drift [92], but a clonal selection during the first engraftment of a human cancer in a mouse is an inevitable consequence of an incompatibility between cytokines produced in the host and the corresponding receptors in the graft. The ligand-receptor incompatibility is a fifth shortcoming that is innate to all xenograft models. Given the importance of IL-6 class inflammatory cytokines in PDAC as discussed earlier, it should be recognized that the mouse LIF and IL-6 do not activate the human receptors [93–95]. Even selected peptide hormones like the mouse prolactin are unable to stimulate the human prolactin receptors [96], and there might be many more examples for ligand-receptor incompatibilities that provide a biological platform for a very efficient clonal selection of cancer cells. Therefore, it may not be surprising that particular molecular subtypes of PDAC such as those with basal-like characteristics may show better engraftment and growth rates or a predominant presence of an activated murine stroma regardless of the molecular characteristics of the implanted primary tumor [9]. It will be interesting to see how these intrinsic limitations, in particular the genetic drift, will impact the rigor and reproducibility of research findings as these models are being propagated in different laboratories using a variety of methods and immunocompromised mouse strains. It seems almost inevitable that PDX models have to come with an “expiration date”. If the contamination and misidentification of the few conventional cell lines was a subject for concern [97, 98] (see the registry of misidentified cell lines of the International Cell line Authentication Committee), then the exponential generation of hundreds and thousands of PDX lines on a global scale and their use without universal standards and controls will most certainly amplify the already exiting issues regarding rigor and reproducibility in cancer research. On a final note, if it is correct that a significant fraction of metastases is monoclonal or polyclonally seeded [99, 100] and that metastases may be genetically distinct from parental clones as a result of sub-clonal evolution [101], then the frequently stated claim that an explant or a PDX model derived from a small tumor fragment of a primary or metastatic site can serve as a “patient avatar” should be taken with caution.

## Early animal models for sporadic pancreatic cancer

Long before the development of genetically engineered mouse models for pancreatic ductal adenocarcinoma, it was observed that albino rats develop pancreatic neoplasms when fed a diet that is supplemented with 2-acetylaminofluorene [102]. However, these tumors were mostly adenomas and acinar cell carcinoma that did not exhibit histopathological features of PDAC. Subsequent studies have shown that more than a dozen chemical compounds can induce pancreatic cancers in various animal models. A detailed description of the biological effects of these agents can be found in a review by Rao [103]. Interestingly, rats and mice seem to develop primarily acinar-type tumors following treatment with different types of chemical carcinogens. An examination of chemically induced tumors in the guinea pig revealed that adenocarcinoma with a duct-like morphology may have originated from acinar cells that underwent acinar-to-ductal metaplasia (ADM), which is now a widely accepted paradigm for the developmental progression of PDAC in genetically engineered pancreatic cancer models. Among the various chemical carcinogen-induced pancreatic cancer models, a notable achievement was the generation of the first mammalian model with *bona fide* PDAC pioneered by Parviz Pour and colleagues in 1974 at the University of Nebraska Medical Center [104] (Fig. 1). This team demonstrated that Syrian golden hamsters continuously treated with 2,2′-dihydroxy-di-N-propylnitrosamine (DIPN) develop pancreatic tumors that resemble human PDAC based on histopathological features. These tumors showed perineural invasion and have the propensity to metastasize to lymph nodes as well as the stomach, liver, and lung. The hamster model also shows clinical features commonly observed in human patients such as weight loss, jaundice, ascites, and thrombosis. A review of the literature on the molecular characteristics of the hamster model revealed that similar to human PDAC, the vast majority of pancreatic tumors in the Syrian golden hamster carry *KRAS* point mutations in codon 12 in addition to deletions and aberrant methylation of *CDKN2A* (for a comprehensive list of references, please refer to Takahashi et al. [105]). However, mutations in *Trp53* have not been reported in the hamster PDAC model. In contrast to the aforementioned guinea pig, Meijers and colleagues were unable to confirm that chemical carcinogen-induced ductal lesions in the hamster developed through ADM [106]. Instead, the authors proposed that premalignant lesions may have originated from ducts or intra-acinar ductal cells. Over many years, the various chemically induced models for sporadic pancreatic cancer have been instrumental to examine risk factors for cancer initiation and biological characteristics [85]. They have provided the first insight into the cellular origins of pancreatic cancer and they laid the groundwork for alternative developmental trajectories of PDAC such as ADM that were subsequently studied in genetically engineered pancreatic cancer models.

## Genetically engineered mouse models of pancreatic ductal adenocarcinoma

### Conventional transgenic mice and spontaneous mutants expressing oncogenic KRAS

Major technological advances in the 1980s and 1990s made it possible to generate a myriad of genetically engineered animal models to study the roles of individual genes in developmental processes in vivo. Transgenic insertional mutagenesis and the targeted manipulation of endogenous loci in embryonic stem (ES) cells were employed to create mouse models for specific tumor types including pancreatic cancer. Given the frequent occurrence of activating mutations in *KRAS* in human pancreatic cancer, the first efforts of modeling PDAC in mice concentrated on the targeted expression of oncogenic KRAS to the exocrine pancreas. At the same time, experimental studies were conducted to assess the biological consequences of the spontaneous activation of endogenous mutant *KRAS* alleles. Grippo and coworkers generated transgenic mice that expressed the coding exons of mutant Kras^G12D^ under the control of the human elastase (Ela) promoter/enhancer [107]. The acinar cell-specific expression of mutant KRAS in aging Ela-Kras^G12D^ transgenics led to the initiation of preinvasive pancreatic neoplasia with duct-like morphologies. The collective results of this study demonstrated that gain-of-function mutations in KRAS play a crucial role in the initiation of pancreatic neoplasms, but mutant KRAS alone was insufficient to drive invasive characteristics of transforming acinar cells and their duct-like descendants. Using a cytokeratin 19 (CK19) reporter transgene, this work provided supporting evidence that KRAS-induced duct-like lesions may occur through ADM or the transformation of centroacinar cells. It is interesting to note that the targeted expression of oncogenic KRAS to CK19-positive epithelial cells in transgenic mice did not cause pancreatic tumors [108], which may suggest that the pancreatic ductal epithelial cells may not be significantly more susceptible to KRAS-induced neoplastic transformation. A noticeable phenotype in the pancreata of aging CK19-KRAS^V12^ transgenics was the infiltration of lymphocytes around the ducts and occasionally small hyperplastic regions.

To gain first insight into biologically relevant functions of mutant KRAS expressed from its endogenous locus, Johnson and coworkers at the Massachusetts Institute of Technology utilized gene targeting and insertional mutagenesis in ES cells to generate mouse strains that carry alleles of oncogenic *Kras* that are somatically activated in many tissues and cell types through spontaneous recombination events, i.e. intrachromosomal recombination or unequal sister-chromatid exchange [109]. These mutant mouse lines exhibited an extensive tumor burden around 200–300 days of age. While the lung was the most frequent site of tumor occurrence, the KRAS mutants also developed thymic lymphoma and skin papillomas, but pancreatic neoplasms were not observed. There have not been any follow-up studies to shed light on potential mechanisms, but it seems plausible that variations in neoplastic progression among these organs are a combined consequence of differences in normal tissue homeostasis (e.g., proliferation, tissue renewal), rates of spontaneous recombination, and essential contributions of inflammation-induced processes (e.g., ADM following pancreatitis).

### KC and KPC models

Based on experiences with the spontaneous KRAS tumor models, there was a need to apply alternative approaches to express mutant KRAS from its endogenous locus specifically in pancreatic progenitors and their more differentiated descendants in the exocrine pancreas. A suitable approach was the Cre/*lox*P recombination system [110], which had been previously used to study the biological roles of oncogenes and tumor suppressor genes in other organ systems [111, 112]. The successful establishment of Cre/*lox*P-based pancreatic tumor models that express oncogenic KRAS predominantly in the exocrine pancreas was aided by two crucial technological advances: (1) the creation of the *Kras*^*LSL−G12D*^ knockin line [113], which carries a transcriptional *STOP* sequence flanked by two *lox*P sites in front of the G12D mutant coding exon, and (2) the development of strains that express Cre recombinase under the control of promoters/enhancers of the *Ptf1-p48* and *Pdx1* genes [114, 115]. Seminal work conducted by several research teams had previously discovered the expression of the transcription factors *Ptf1-p48* and *Pdx1* in common endocrine/exocrine precursors and their essential roles in early pancreatic development [116–118]. Hingorani and coworkers crossed the Cre strains with *Kras*^*LSL−G12D*^ knockin and observed that the pancreas-specific expression of mutant KRAS led to the development of PanIN lesions that infrequently progressed to invasive adenocarcinomas [115]. It is interesting to note that mutant KRAS-expressing cells are already present at birth since the p48-Cre and Pdx1-Cre transgenes are active during embryogenesis and delete the *STOP* sequence in the *Kras*^*LSL−G12D*^ allele. However, the formation of preneoplastic changes occurs postnatally. Similar to the Ela-KRAS transgenics [107], the Pdx1-Cre *Kras*^*LSL−G12D*^ double mutants (often referred to as the “KC model”) provided evidence for a pivotal role of mutant KRAS in the formation of PanIN lesions that have the propensity to progress into invasive PDAC. Genetic crosses of the KC model with *Cdkn2a-*deficient mice or a mutant *Trp53* line (referred to as the “KPC model”) firmly established the biological significance of these tumor suppressor genes in PDAC progression and metastasis [115, 119, 120]. These models resemble the most frequently occurring human pancreatic adenocarcinoma subtypes on the histopathological level, including the formation of a dense tumor-associated desmoplastic stroma. The KC and KPC models are now considered the “gold standard” for genetic experiments to study the roles of molecular pathways in PDAC initiation and progression (Fig. 1). There are several excellent reviews that describe examples of how the KC and KPC mice have been applied to study the biology of pancreatic cancer and the significance of genes and molecular pathways in cancer initiation [121, 122]. Looking ahead, a definitive classification of the molecular subtypes might be needed in the future to better characterize the existing and newly generated genetically engineered tumor models to establish relevance to the molecular subtypes within the human disease spectrum. It is also evident that many published studies describing genetic experiments with the KC or KPC models lack a thorough analysis of the function of the genes of interest in normal pancreatic development. Developmental defects can greatly alter the onset of tumor development. In the worst-case scenario, the Cre-mediated deletion of a gene of interest might lead to a negative selection of knockout cells, thereby eliminating the pool of cells that express mutant KRAS, which can profoundly affect the interpretation of the results.

### Tetracycline-controlled expression models

The KC and KPC mice are excellent research tools to initiate the sporadic formation of pancreatic tumors, but these models are less suitable to investigate the continued significance of KRAS in tumor maintenance and progression. In the genomics era where mutations are often given a perceived importance in carcinogenesis or therapy just based on their frequent occurrence, it is necessary to reemphasize that not all mutated genes function as drivers of the tumorigenic process. Since the seminal work by Ewald and colleagues [123] in the mid-1990s, it is known that even a potent transforming oncogene like the SV40 large T antigen can become a passenger that is no longer essential for the maintenance of late-stage cancers. Under specific conditions such as the maintenance of human and mouse pancreatic cancer cells in culture, this is also true for mutant *KRAS*, which is not strictly required for cancer cell survival in vitro as long as these cells also possess a wild-type *KRAS* allele [124–126]. To discriminate the biological significance of oncogenic KRAS in cancer initiation versus progression in vivo, several laboratories generated transgenic mice that express mutant forms of KRAS in a tetracycline-controlled manner in specific tissues [127–129]. The lines expressing the mutant KRAS^G12D^ in a ligand-dependent manner have been subsequently applied to suppress oncogenic RAS signaling in established pancreatic cancers, which led to the regression of primary and metastatic tumors in vivo [125, 130–132]. Our team also developed a pancreatic cancer model with a targeted expression of the c-MYC oncogene in pancreatic progenitors under the control of the tetracycline-responsive transactivator [17]. As mentioned in the previous sections of this review, the overexpression of c-MYC was sufficient to initiate preneoplastic lesions with duct-like morphology that swiftly progressed into poorly differentiated tumors with a high propensity to metastasize. Similar to the mutant KRAS models, the survival of pancreatic cancer cells at primary and metastatic sites was dependent on the perpetual expression of c-MYC. Despite the macroscopic regression of tumors following the ablation of the oncogenic drivers in all these genetic models, a significant number of dormant cancer cells remained that caused a swift recurrence of the tumors following the reactivation of the corresponding oncogene [17, 132]. Hence, a significant value of the tetracycline-controlled expression models for pancreatic cancer is the ability to study the biological processes and molecular mechanisms that mediate tumor cell dormancy. Using the reversible c-MYC cancer model, our team found that the dormant cell population contained a significantly higher number of cells that express markers associated with stemness [17]. The survival of dormant cancer cells might be upheld by the persistent presence of the tumor-associated stroma, which did not undergo a significant remodeling process despite the absence of most tumor cells and the induction of autophagy during tumor regression. These observations from the c-MYC tumor model were later validated in a study by Viale et al. [133] in a mutant KRAS-dependent cancer model, and this team reported a potential role for mitochondria in cancer cell survival following the downregulation of mutant KRAS. In a subsequent line of investigation, our team identified an increase in IGF1 autocrine signaling as a common mechanism for the survival of dormant cells in vivo in the absence of oncogenic KRAS and c-MYC [125]. The pharmacological inhibition of IGF-1R signaling led to a substantial eradication of residual disease and a significant delay in cancer recurrence in response to the reactivation of KRAS or c-MYC. Following an extended latency, a subset of quiescent pancreatic cancer cells can emerge from dormancy and lead to cancer relapse without re-expression of oncogenic KRAS. A suggested mechanism by which cells can bypass their initial dependency on mutant KRAS for cell proliferation is the amplification or overexpression of the transcriptional coactivator YAP1 [134, 135].

In summary, the tetracycline-controlled expression models for pancreatic cancer have been instrumental for the identification of cellular processes and molecular pathways by which a subset of cancer cells can escape a targeted ablation of KRAS and its downstream effector c-MYC. A discussion of the implications of these findings for the future development of targeted therapies to treat PDAC can be found in a review by Lin et al. [136]. At present, the findings from the tetracycline-controlled PDAC models may not seem significant for clinical translation due to the lack of pharmacological agents that target the KRAS^G12D^ mutant form. Given the recent approval of sotorasib to treat certain KRAS^G12C^-driven lung cancers, however, targeting mutant KRAS may no longer seem to be an unattainable goal. Identifying the mechanisms by which cancer cells bypass their dependence on mutant KRAS may aid the development of treatment strategies that increase the efficacy of KRAS inhibitors [124].

### Application of genetic models to uncover the contribution of specific mutations to different developmental trajectories towards pancreatic cancer

Although PanINs are considered to be the common precursors of PDAC, a subset of cystic lesions may develop into invasive cancer, in particular intra-ductal papillary mucinous neoplasm (IPMN) and mucinous cystic neoplasms (MCN). A recent review by Sethi et al. provides a detailed overview of the classification of pancreatic cysts, the pathogenesis and occurrence of genetic alterations, and the differences in the propensity of these preneoplasms to progress into PDAC [137]. This article also provides information about genetically engineered mouse models that resemble specific histopathological subtypes of pancreatic cysts in humans. Genome-wide sequencing studies have revealed that mutations in *GNAS* and/or *KRAS* are specifically associated with the formation of IPMNs [44, 138], and gain-of-function mutations in *GNAS* are present in a subset of PDACs [13, 15]. Several research teams generated genetically engineered mouse models that express gain-of-function mutations of GNAS (R201H and R201C) to address the biological significance of GNAS alone or in combination with oncogenic KRAS in the formation of precursor lesions as well as the progression and maintenance of pancreatic cancer [139–141]. The collective findings from these models revealed that expression of mutant GNAS alone may lead to low-grade IPMN after an extended latency period, but a gain-of-function in GNAS is insufficient to initiate pancreatic tumorigenesis. However, GNAS^R201H^ and GNAS^R201C^ greatly accelerated the onset of IPMN in the presence of mutant KRAS without frank tumor formation [139–141], and in response to the loss of p53, GNAS/KRAS-double mutant neoplasms swiftly progress to invasive PDAC [141]. By expressing exogenous mutant GNAS in a tetracycline-controlled manner in established KRAS-mutant tumors, Ideno et al. [140] and Patra and coworkers [141] were able to delineate the specific functions of GNAS in tumor cell differentiation and cancer maintenance. Ideno et al. [140] reported that expression of GNAS^R201C^ led to more differentiated KRAS-associated tumors through alterations in HIPPO signaling. The study by Patra and coworkers [141] provided important evidence that the same mutation in GNAS was critical for tumor maintenance despite the persistent activation of oncogenic KRAS and mutant p53. This observation suggested that cooperating gene mutations may orchestrate heterogeneous molecular circuits that fuel the growth of KRAS-mutant pancreatic tumors and may provide new avenues for targeted therapies. Both studies by Patra et al. and Ideno and colleagues exemplify the value of combining different genetic model systems to uncover unique molecular and biological processes in a rigorously controlled manner in vivo.

### Advanced approaches in genetic cancer modeling

A review of technologies to genetically alter the genome to study pancreatic cancer development would not be complete without mentioning recent advances in the generation of models that utilize multiple recombinases as well as the applicability of CRISPR/Cas9-based gene editing. To experimentally assess the significance of a certain gene for cancer prevention or therapy, it might be necessary to delete a gene of interest at specific stages of neoplastic progression (e.g., PanINs versus established tumor). In the KPC model, a target gene is typically deleted before or during the first activation of oncogenic KRAS. A delay in tumor formation in response to the knockout of a gene of interest in the KPC model should never be viewed as evidence that this gene is a genuine candidate for therapy. Also, such a finding should not be considered proof that this gene plays a role in cancer prevention unless it was validated that a Cre-mediated conditional knockout of the target gene does not lead to a negative selection of pancreatic progenitors and their descendants with mutant KRAS. Schönhuber and colleagues [142] recently developed a next-generation dual-recombinase system where the oncogenic activation of KRAS is uncoupled from the conditional deletion or activation of a target gene. In this model, a gene of interest can be deleted before, during, or after the formation of pancreatic cancer. In a nutshell, a Pdx1 promoter-driven Flp recombinase transgene (Pdx1-Flp) deletes a transcriptional *STOP* sequence that is flanked by two *Frt* sites (*FSF* sequence) in front of the G12D mutant coding exon of the *Kras*^*FSF−G12D*^ knockin allele [143]. Simultaneously, the Flp recombinase also excises the *FSF* sequence in the *Rosa26*^*CAG−FSF−CreERT2*^ knockin transgene that, in turn, expresses a tamoxifen-inducible Cre recombinase (Cre^ERT2^). Technically, a Pdx1-Flp *Kras*^*FSF−G12D*^* Rosa26*^*CAG−FSF−CreERT2*^ mouse co-express the mutant KRAS and the Cre^ERT2^ in a constitutive manner when they are born. The Cre^ERT2^ may then facilitate the deletion of the conditional knockout alleles of a gene of interest (e.g., *Trp53*^*flfl*^) when the mice are being treated with tamoxifen [142]. Hence, the temporally and spatially controlled deletion of a gene relies on the sequential activity of two recombinases (Flp and Cre^ERT2^), while the initiation of mutant KRAS is solely dependent on Flp. Although this approach requires a more extensive breeding scheme using multiple mouse strains, the genetic model generated by Schönhuber and colleagues holds great promise to conduct biologically relevant studies that address the function of genes at defined developmental stages in a very rigorously controlled manner.

A disadvantage of using genetically engineered strains is the costly maintenance of individual transgenic and knockout lines for ongoing and future studies. Advances in CRISPR/Cas9-mediated gene editing may provide some advantages for generating animal models that sporadically develop tumors in response to the somatic manipulation of endogenous tumor susceptibility genes. In a proof-of-principle experiment, Platt et al. [144] developed an adenovirus-associated vector (AAV) that delivered the guide RNAs to simultaneously knockout *Trp53* and *Stk11* along with a gRNA and homology-directed repair template to introduce a G12D mutation in *Kras*. The vector also contains an expression cassette for Cre recombinase, which induces the conditional activation of a Cas9-expressing transgene that is essential for the targeted manipulation of the three cancer-promoting target genes. The intranasal and intratracheal delivery of the AAV vector into *Rosa26*^*CAG−LSL−hSPCas9*^ knockin mice resulted in the formation of lung cancers within 2 months. While the inducible expression of Cas9 in the mouse is a necessary fail-safe mechanism for the use of an AAV-based gene editing vector, any infected cell within a particular tissue, including stromal cells or lymphocytes, could be subject to the targeted gene manipulation. The success of this methodology relies largely on the biological selection of transformed cells that express mutant KRAS in the absence of one or both tumor suppressors. An adaptation of this approach to model pancreatic cancer would require several changes, including the targeted expression of Cas9 to the exocrine pancreas. Although the simultaneous delivery of gRNAs to manipulate multiple genes reduces the number of breeding steps, it should be recognized that the CRISPR/Cas9-based approach developed by Platt et al. [144] has the same inherited limitations as the KPC model or conventional transgenics in terms of their inability to discriminate the function of a gene of interest during tumor onset, maintenance, and progression.

## Concluding remarks

Over the past two decades, there has been major progress in the generation and analysis of models for pancreatic adenocarcinoma. In addition to the new patient-derived organoid cultures and xenografts, there have been noticeable advances in genetically engineered animal models. In particular, the generation of the KC and KPC models created a lot of excitement [145], and since then, these mice have become a gold standard for mechanism-oriented research studies. Newer models that express cancer-initiating oncogenes, such as KRAS and its downstream effector c-MYC in a ligand-controlled manner, provided additional insight into the significance of these oncogenic drivers in premalignant lesions as well as primary and metastatic tumors in vivo. Moreover, they have been instrumental in defining specific mechanisms for tumor cell dormancy and cancer recurrence in the absence of oncogenic KRAS and c-MYC. These findings can be expected to gain significance as soon as new and improved pharmacological agents are being applied to target specific mutants of KRAS and their downstream targets. The recently developed genetic models that allow the conditional deletion of genes at particular stages of tumor progression will aid the identification of cancer cell-intrinsic mechanisms that orchestrate the growth and survival as well as cellular characteristic that define the molecular subtypes of pancreatic cancer.

Although the molecular determinants for PDAC are not as diverse as in other malignancies such as breast cancer, adenocarcinomas in the pancreas are heterogeneous in their histopathology, gene expression profiles, and composition of the tumor microenvironment. Like any model, the scientific reagents and tools to study pancreatic cancer (i.e., cell lines, organoids, PDX, genetically modified animal models) reflect only certain aspects of the phenotypic and molecular spectrum of the malignancy. Therefore, a single model should never be branded as “authentic” in the sense that it reflects all characteristics of the disease. In reality, however, the perceived authenticity of a model often rests on a limited number of supporting arguments while imperfections are being downplayed or omitted. Opposing viewpoints often originate from a general antipathy against particular experimental tools, and the popularity of certain models is also influenced by patient advocates and funding agencies. Under the auspices of the Human Cancer Models Initiative at NCI, the “next-generation cancer models” are primarily defined as patient-derived organoids and xenografts. Given the innate deficiencies of all xenograft models that were discussed in this review, it is evident that patient-derived tissue transplants and explants will have a limited applicability for mechanism-oriented research. The exponential increase in the number of these specimens along with the lack of universal standards for their use and analysis (e.g., site of implantation, type of genetic host, processing and storage conditions of specimens) will create additional challenges for rigor and reproducibility in biomedical research. It is warranted to discuss whether the term “cancer model” should apply to patient-derived specimens that are not generally available to the research community and those that cannot be authenticated in the laboratories. In general, the selection of appropriate models and methodologies should be guided by the scientific questions and a quest to generate reproducible research data, and to a lesser extent from the viewpoint of the perceived significance of a project or a personal preference of one type of model over another.
